# Clinical forensic medicine in emergency departments: a pilot study of a forensic training and evaluation of its effectiveness in an Italian hospital

**DOI:** 10.1007/s00414-024-03313-0

**Published:** 2024-08-27

**Authors:** Stefano Tambuzzi, Cecilia Rossi, Donatella Pavanello, Riccardo Primavera, Giorgio Costantino, Cristina Cattaneo

**Affiliations:** 1 Istituto Di Medicina Legale, Dipartimento Di Scienze Biomediche Per La Salute, Università Degli Studi Di Milano, Via Luigi Mangiagalli, 37, 20133 Milan, Italy; 2 Fondazione IRCCS Ca’ Granda, Ospedale Maggiore Policlinico, Milan, Italy

**Keywords:** Clinical forensic medicine, Violence by others, Emergency departments, Forensic training, Forensic in hospitals

## Abstract

Because emergency departments are often the first point of contact for victims of violence, it is critical to provide the appropriate treatment in compliance with all necessary medicolegal precautions. For this reason, a randomized controlled trial was conducted at the Policlinico Hospital of Milan (Italy) in which an intervention group (12 physicians) received a 6-h course on clinical forensic medicine and their performance in medicolegal procedures in claimed cases of violence was compared with that of a control group (13 physicians) by means of a 16-item assessment scale over the 3 months before and the 3 months after the course. Overall, 195 medical records were included in the statistical analysis. Out of these cases, 105 occurred before the course (60 analyzed by the control group and 45 by the intervention group) and 90 occurred after the course (45 analyzed by the control group and 45 by the intervention group). The results showed that the overall mean score of physicians who participated to the course increased from 14.0 (IQR 7.0) to 19.0 (IQR 8.0) with a *p*-value < 0.0001 and that the comparison between the intervention group and the control group after the course was 19.0 (IQR = 8.0) and 14.0 (IQR = 7.0), respectively, with a *p*-value < 0.0001. The improvement was very little and below the expectations pointing out that educational courses, although they can be a first step towards raising the ED physicians’ awareness of clinical forensics, may not be enough and that more structured training and new strategies should be implemented.

## Introduction

Interpersonal violence is defined as violence perpetrated by another person or a small group of people. Each type of violence can have different manifestations, that often overlap: physical, sexual, psychological, or caused by deprivation or neglect [1]. Currently, interpersonal violence accounts for a significant proportion of the global burden, with approximately 1.6 millions of deaths every year, with most deaths occurring in low- to middle-income countries [1]. However, the number of deaths is only the tip of the iceberg, as the number of nonfatal casualties associated with violence is enormous, and it is responsible for significant long-term public health consequences at the physical, psychological, social, and economic levels [2].

Every year, millions of people are admitted to Emergency Departments (ED) due to interpersonal violence, and ED health care professionals are often the first to encounter the victims. Therefore, they play a critical role in detecting, recognizing, treating, and especially preventing violence. Unfortunately, they are still inadequately prepared to properly treat violence on the same level as any other disease, as evidenced by the sparse publications on the subject [3, 4]. Two studies conducted by Vieira et al. in Brazilian hospitals [3] and Othman et al. [4] in Malaysia, respectively, show that health workers do not recognize the prevalence of the problem, are unconfident to ask about violence, and are not efficient enough to deal with it. Their knowledge of the procedures required to properly document, collect, and preserve forensic evidence was also inadequate [3, 4]. In addition, a recent study by Walz et al. [5] comparing forensically relevant aspects of clinical forensic examination of victims of physical and sexual violence by clinicians and forensic examiners found a statistically significant difference between the two groups, mainly caused by improper assessment by clinicians. Similar findings resulted from the ED of a I-level trauma center in California [6] The picture is also quite bleak in Italy, as shown by a recent publication reporting on a study conducted at the ED of the Policlinico Hospital of Milan (in northern Italy) [7]. In most cases all the measures with medico-legal implications were neglected by the ED physicians, resulting in a loss of data not only for the judiciary, but also for the correct interpretation of what happened. While this may seem directly interpreted as a service to justice, in the end, it is always related to the protection of the victim’s health. Indeed, the collection of data and evidence is closely related to medical acts [6]. It seems clear, therefore, that emergency room physicians, are in the perfect position to gather forensic evidence and data in a timely manner to ensure continuity "from trauma to trial", thereby achieving both justice and health protection goals [8–10]. In this scenario, health professionals working in primary care, should be trained to acquire specific forensic skills to better assess and evaluate injuries resulting from violence and to improve the network between physicians, social services, and the territorial institutions that help victims.

For this reason, it was considered appropriate to conduct another study at the emergency department of the Policlinico Hospital of Milan, where the implementation of medico-legal procedures had already been found to be deficient in a previous work [7]. A group of physicians participated in a short (6-h) but intensive course on clinical forensic medicine, and their performance was compared with that of a group of physicians who had not participated in the course. The aim was to determine whether the course had affected the physicians’ behavior in daily practice by improving compliance with medicolegal procedures and if there had been a change in the way violence was handled.

## Material and methods

### Setting and study design

A randomized controlled trial was conducted at the ED of the Policlinico Hospital of Milan, one of the most important hospitals in Lombardy (northern Italy), located in the city center with almost 60.000 ED admissions per year (excluding pediatrics and gynecological), out of which about 1.000 due to interpersonal violence [7]. All 25 emergency physicians working here were included in this study at the beginning of April 2022. Of these, 12 physicians (intervention group) were randomly selected to participate in a 6-h training course on clinical forensic medicine taught by two forensic pathologists. The remaining 13 physicians were assigned to the control group, which did not participate in the course. The randomization was performed by drawing casually the physicians working in the ED at the beginning of April 2022 from a pool of names in a basket; alternately one was assigned either to the control group or to the intervention group. Residents and surgeons were not selected for the study because the former are still in training and the latter intervene when the patient requires immediate emergency surgery.

The topics covered in the course focused mainly on the different types of interpersonal physical violence injuries, their evaluation and documentation, including the proper tests and imaging procedures to perform in the different scenarios. Special emphasis was placed on interviewing the patient for important information related to the aggression, taking photographs of the injuries, collecting samples, and a need for a standardized procedure for reporting and describing injuries (body site, number of lesions, size, color and shape).

After the course, a smartphone was made available to take photographic documentation. Swabs and a reference scale for photographs were also provided to staff. To protect patients’ privacy, the smartphone was kept in a safe place, and physicians were instructed not to use personal devices to take photographs. In addition, each photograph had to be labelled with the patient's medical record number without adding sensitive patient’s information.

Subsequently, all medical records of patients admitted at the ED from January 2022 to June 2022 and labeled by health professionals as “patient victim of violence by others” were extrapolated in a completely anonymous way. This time frame of 6 months was chosen to include 3 months before and after the training course. The extrapolated records may have involved non-sexual physical assault as well as sexual assault: the study assessed only the performance of clinicians at the ED to describe, evaluate, and treat violence, regardless of the fact that some of these victims might later be referred to a specialized sexual violence center.

A 16-item scale was developed by the two forensic experts who conducted the course to evaluate the selected medical records. The items were divided into 6 main categories: history taking, injuries description, sample collection, imaging prescription and reporting to the judicial authority. This last category has been added because in Italy there are numerous crimes that it’s mandatory to report to the judicial authorities (depending on their codification and seriousness) regardless of the will of the victim. It was therefore important to include also this category to assess if the clinicians were aware of their legal duties.

Depending on the accuracy of the physicians’ behavior in the above-mentioned tasks, each item was scored and each case was assigned a total score from 0 to 41, according to Table 1. It is important to note that medical records that contained a history suggestive of aggression or violence but for which any evidence of trauma were specifically excluded after a full-body examination were not scored and were excluded from the analysis. This avoided assigning a score that was not representative of the physician's correct behavior.

**Table 1:** The 16-item scale used for scoring extrapolated medical records

Main categories		Score Assessment			
		Absent	Superficial	Acceptable	Excellent
1. History taking	When (period)	0	1	2	3
	Who (violent subject)	0	1	2	3
	Where (setting)	0	1	2	3
	How (dynamic of the facts)	0	1	2	3
2. Description of the lesion	Type	0	1	2	3
	Dimensions	0	1	2	3
	Color	0	1	2	3
	Shape	0	1	2	3
	n° of lesions	0	1	2	3
	Site	0	1	2	3
	Manner	0	1	2	3
	Aging	0	1	2	3
3. Photographs of the lesion		Not done = 0	Done but without measure references = 1	Correctly done = 2	
4. Sample collection (residues/swabs)		Not collected = 0		Collected = 1	
5. Imaging tests (X-rays, CT scan, MRI, ultrasonography)		Not performed = 0		Performed = 1	
6. Report to the judicial authority		Not done = 0		Done = 1	
Total Score				Min. 0—Max. 41	

Each item was assigned a variable score depending on the accuracy of the information provided (0 to 3 for description of injuries and aggression) or the procedures performed (0 to 2 for taking photographs and 0 to 1 for collecting samples, performing imaging, and reporting to judicial authority)

Finally, all selected medical records were evaluated to assess the performance of the ED physicians and to determine whether the training course had affected their behavior in daily practice by improving compliance with medicolegal procedures. Indeed, the primary outcome of the study was the change in the median total score of the two groups before and after the course. It is crucial to point out that all physicians who participated in the course were unaware that their performance would be evaluated in a second period. The assessment of each case was initially done blindly, i.e., without the knowledge of the physician who wrote the medical record; this procedure avoided bias in the evaluation. Only in a second time, each medical record was matched to the physician who completed it to determine whether he or she participated to the course.

### Statistical analyses

A comparison of the scores collected in the 3 months before and in the 3 months after the training was made for both the intervention and control groups, to determine whether there had been a change in the way violence was handled. Statistical analyses were performed for this purpose, in particular the tests have been carried out using the software IBM SPSS Statistics 29.0.1.0 (IBM, New York, USA) [11]. Descriptive data were reported as median and interquartile range because their distribution was not normal. A nonparametric test, the Wilcoxon rank-sum test, was used to compare groups of data with continuous variables[12]. In detail, the intervention and control groups data were furtherly divided into two subgroups, the pre-course group and the post-course group, resulting in a total of four subgroups: pre-course participants, post-course participants, pre-course non-participants, and post-course non-participants. The above test was used to compare the differences in median scores between these 4 groups. For a more comprehensive assessment, a sensitivity analysis was performed to determine whether the result changes if the categories "sample collection", "imaging performance" and “report to the judicial authority” are removed from the total score assessment, as the need for these measures may depend on the individual case and they are not always necessary.

In addition, a statistical analysis was carried out for each of the six main categories to check whether there was a correlation between attendance at the course and completion of the tasks (history taking, lesion description, photographic documentation, samples and swabs collection, imaging performance and report to the judicial authority). To this end, a chi-square test was used to compare the number of tasks performed by the control and intervention groups in the post-course period, and a Fisher’s exact test was used in cases where the expected values were below 5 [13]. A *p*-value < 0.05 has been considered a significative statistical difference.

Finally, since the access of victims of violence to the ED is an unpredictable variable, the sample size was not calculated. However, as this is a pilot study, a period of 6 months (3 months before and 3 months after the course) was considered appropriate, as around 60.000 people come to the ED every year. In addition, this short-term period was considered appropriate also to avoid confounding factors (i.e. physician turnovers or organizational changes) and, on the other hand, a flattening of the skills acquired during the course.

## Results

The intervention group (12 ED physicians) was composed of 4 males (33%) and 8 females (67%); the overall mean age was 35.5 years (IQR = 37 – 33.75), and the median years since master’s degree was 9.5 years (IQR = 11 – 7.75). The control group (13 ED physicians) were composed of 7 males (54%) and 6 females (46%); the overall mean age was 33 years (IQR = 37 – 31) and the median years since master’s degree was 7 years (IQR = 11 – 5).

**Fig. 1:**
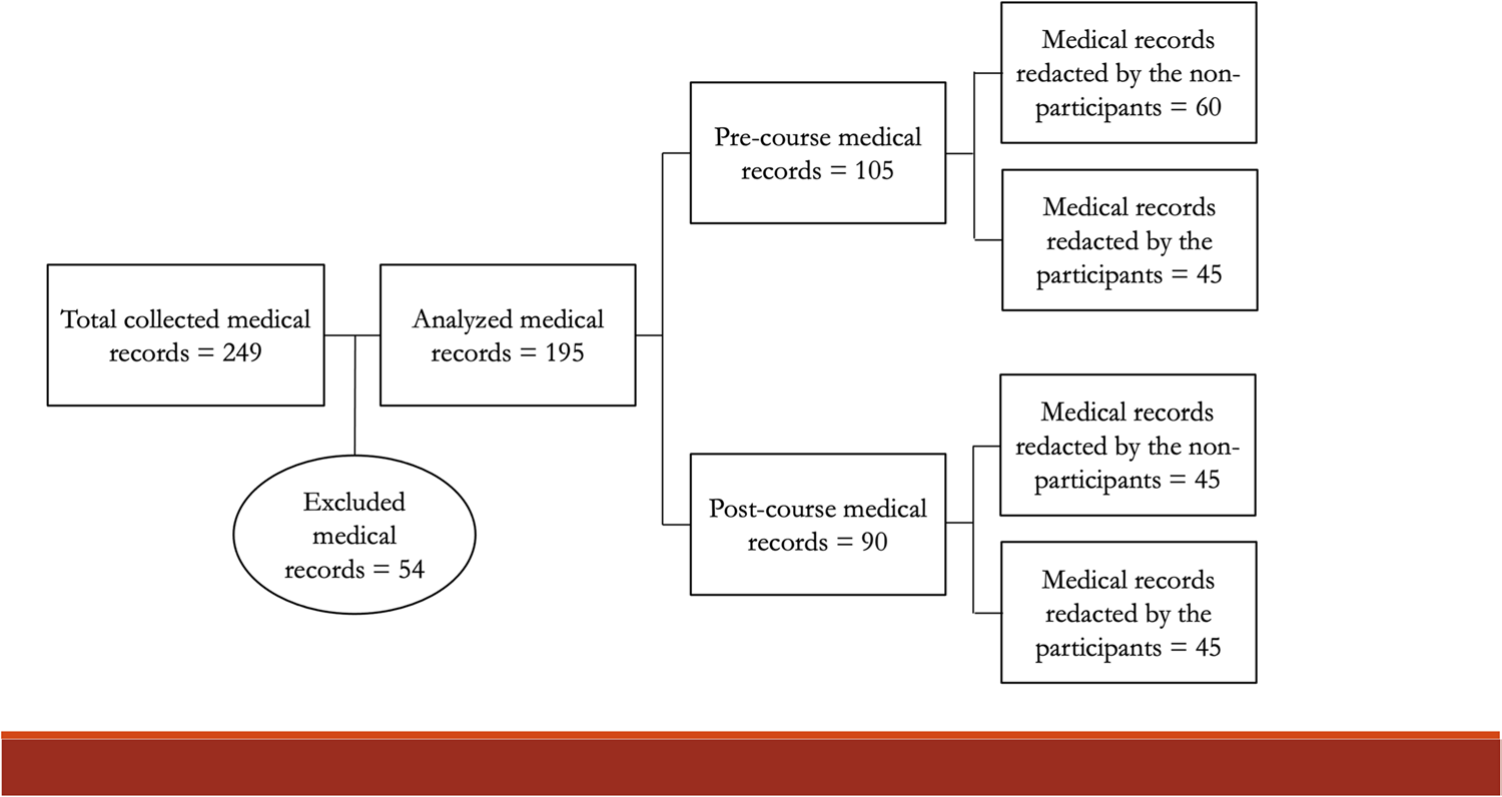
Flow chart of the selected cases

From January 2022 to June 2022, 249 medical records of patients who claimed to have been victims of violence by others were extrapolated. Out of the total, 54 were excluded because 33 cases had been handled by residents or surgeons, and in 21 cases the presence of injuries had been specifically excluded after a full-body examination. Finally, 195 medical records were included in the statistical analysis. In detail, there were 105 cases before the course—60 from nonparticipants and 45 from participants -, and 90 after the course—45 from nonparticipants and 45 from participants -, as shown in Fig. 1.

The characteristics of the patients in the analyzed medical records and the type of trauma to which they were exposed are reported in Table 2. Table 3 shows the absolute number of tasks performed or not performed by the control and intervention groups in the period after the course. In the three cases only, in which a photograph of the injuries had been taken, a check between the photographed injury and the description by the clinician was made. In all three cases, the description proved to be correct.

**Table 2:** Epidemiological characteristics of the subjects analyzed in the study with details of the injury mode

Category	Variable	*N* (%)
Sex	Male	129 (66.2)
	Female	64 (32.8)
	Not reported ¥	2 (1)
Age	18–29	76 (38.9)
	30–39	42 (21)
	40–49	32 (16.4)
	50–59	27 (13.8)
	60–69	15 (7.7)
	70–79	1 (0.5)
	80–89	1 (0.5)
	Not reported ¥	2 (1.0)
Injury mode	Blunt force trauma	166 (85.1)
	Stab wounds	9 (4.6)
	Bite marks	4 (2.1)
	Mechanical asphyxia	3 (1.5)
	Sexual violence	2 (1.0)
	Not reported ¥	11 (5.6)

¥ The information was not reported in the analyzed medical records

Total selected population (*N* = 195)

**Table 3:** Number of forensic tasks performed or not performed by the two groups in the period after the course

Post-course period		Performed	Not performed
History taking	Participants (*n* = 45)	45	0
	Non-participants (*n* = 45)	45	0
Lesion descriptions	Participants (*n* = 45)	37	8
	Non-participants (*n* = 45)	33	12
Photographic documentation	Participants (*n* = 45)	3	42
	Non-participants (*n* = 45)	0	45
Sample and swabs collection	Participants (*n* = 45)	0	45
	Non-participants (*n* = 45)	0	45
Imaging performance	Participants (*n* = 45)	38	7
	Non-participants (*n* = 45)	42	3
Report to the judicial authority	Participants (*n* = 45)	37	8
	Non-participants (*n* = 45)	27	18

Overall, it was observed that for the 60 cases belonging to the pre-course control group the median score was 14 (IQR = 15.5–10.0 = 5.5) compared to the median score of 14 (IQR = 17.0–10.0 = 7.0) obtained for the 45 cases belonging to the pre-course intervention group (*p*-value = 0.6362). No difference was observed between the behavior of physicians in the two groups at baseline (before the course). Instead, it was observed that for the 45 cases belonging to the post-course control group the median score was 14 (IQR = 17.0–10.0 = 7.0) compared to the median score of 19 (IQR = 24.0–16.0 = 8.0) obtained for the 45 cases belonging to the post-course intervention group (*p*-value < 0.0001).

**Fig. 2:**
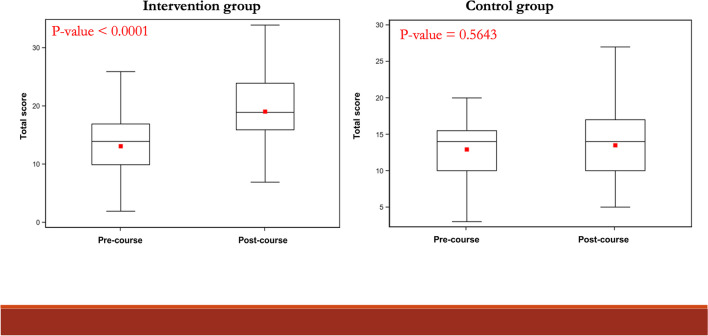
Box-plots of the total score variation in the intervention and control groups before and after the course

Moreover, the median score obtained by the pre-course control group was 14 (IQR = 15.5–10.0 = 5.5) compared to the median score of 14 (IQR = 17.0–10.0 = 7.0) obtained by the post-course control group (*p*-value = 0.5643). No difference was observed between the physicians’ behavior who did not participate in the course, as graphically shown in the boxplot reported in Fig. 2. Instead, the median score obtained by the pre-course intervention group was 14 (IQR = 17.0–10.0 = 7.0) compared to the median score of 19 (IQR = 24.0–16.0 = 8.0) obtained by the post-course intervention group (*p*-value < 0.0001). A statistically significant difference was observed between the physicians’ behavior who participated in the course. Graphically, this significant difference can be seen in the boxplot shown in Fig. 2.

The sensitivity analysis, in which the categories that could depend on the individual case (“sample collection", "imaging performance" and “report to the judicial authority”) were removed from the total score result, showed no difference from the main analysis where they were included in terms of statistical significance. In fact, even in this case, a statistically significant difference was observed in the median score values between the control and intervention groups in the post-course period (*p*-value < 0.0001) and in the median score values of the intervention group in the comparison between the pre- and post- course periods (*p*-value < 0.0001).

Finally, regarding the 6 main categories, the statistical analysis revealed a correlation between participation in the course and the fulfilment of forensic tasks as far as the category “report to the judicial authority” is concerned (*p*-value of 0.02). No correlation was found for the other 5 categories.

## Discussion

Considering the importance of clinical forensic interventions in emergency departments, a study was conducted to determine whether a short (6-h) but intensive course could have an impact on physicians’ behavior in daily practice by improving adherence to forensic medicine procedures and whether there was a change in the way they dealt with violence. The analysis of the physicians’ demographic characteristics revealed that the two groups had very similar features in term of average age, gender, and years since completing the master’s degree, suggesting that there was no professional bias in the assessment of patients with violence-related injuries. A comparison was made between the intervention and control groups before and after the course. Overall, some differences were found. In the intervention group, all the single items were quite poor at the beginning of the course, whereas after the course, there was higher compliance with forensic medical procedures. Indeed, the only cases in which photos of the injuries were taken after the course occurred within physician belonging to the intervention group. Globally, the comparison of the mean total scores before and after the course within the intervention group showed a statistically significant difference.

Physicians in the control group showed different trends in the three months after the course (in which they had not participated): photographs and swabs/samples were never taken and the comparison of median total scores before and after the course in the control group revealed a non-statistically significant difference (scores remained stable).

It is interesting to note that the intervention and control groups performed quite similarly at baseline (before the course) and that the comparison of the overall scores between the two groups showed no significant difference. Although a significant difference between the two groups after the course was found for the single category of “reporting to the judicial authority”, an overall evaluation of the results of the main study analysis showed that the mean total score of physicians participating in the course increased from 14.0 (IQR 7.0) before the course to 19.0 (IQR 8.0) after the course. In addition, comparison between the intervention group and the control group revealed a difference in the mean total score after the course: 19.0 (IQR = 8.0) in the participant group and 14.0 (IQR = 7.0) in the nonparticipant group. These differences proved to be statistically significant, indicating that the ED physicians who had participated in the course had better compliance with medicolegal procedures. The training has thus helped to provide the ED physicians with new clinical forensic medicine knowledge but it was only partially put into practice, as the medico-legal aspects were still largely neglected. Indeed, considering that 41.0 was the highest possible score that could be achieved, an improved median score of 19.0 and an increase of only 5.0 points is below the expectations and far from being considered a fully achieved goal. Thus, the results of this study show that a simple course can be a first step towards raising doctors’ awareness of clinical forensics, but it was not enough to meet the needs of patients who were victims of violence.

The reasons that led to this small improvement can be many. First, we cannot guarantee that all 12 physicians selected for the intervention group were truly interested and willing to acquire new knowledge and skills. Second, the course had a short duration of 6 h, which obviously does not guarantee complete training and acquisition of all the forensic information and skills. However, it allowed to provide all the basic information and was the maximum time for which it was possible to withdraw 12 emergency physicians at the same time from the needs of the department. Another element that must be considered to interpret the results correctly is that the developed scoring system included some items such as taking swabs/samples, prescribing imaging tests and reporting to the judicial authority that are not always necessary. Swabs should be taken, for example, if residues of organic material may be found in or around the lesion. This is mandatory in cases of sexual abuse or bites, but they may be waived in certain circumstances, such as if the injured body area was covered by clothing or washed [14]. Furthermore, as much as the two groups initially overlapped, the distribution of more severe cases in the selected period of time that would require more attention was an uncontrollable variable, of course. This certainly has implications for taking swabs, prescribing imaging tests, and reporting to the judicial authority. Precisely for this reason, a sensitivity analysis was carried out in which these three categories, which may depend on the individual case, were excluded from the total score, which did not lead to any differences in the statistical interpretation of the results. Indeed, the above-mentioned categories add up to a maximum of 3 points, and even if removed the achievable total score would then be 38 instead of 41. Therefore, it is clear that the central core of the total score is represented by taking the medical history, describing the injuries, and taking photographs, which should have been done in all the cases, but it was not. It is evident that the physicians who participated to the course tried to implement these behaviors, which actually increased the overall score. In particular it has been observed that, regarding the report to the judicial authority, a significant increment occurred in the post-course for the intervention group, implying an increased awareness and attention. This is crucial because it is a legal requirement that Italian doctors must comply with when dealing with a crime that is mandatory to report. Disregarding this specific aspect often leads to non-reporting with a double consequence: the doctor violates a penal law and the patient is not protected. This increased awareness was clearly not present in the physicians who had not attended the course.

However, the main reason for the unsatisfactory results is probably the simple and trivial lack of time, which is the main obstacle to the good will of most physicians. Of course, the main task of emergency room medical personnel is to identify and treat life-threatening conditions. However, even when this task is accomplished or the conditions are not life-threatening, not all forensic techniques and procedures learned in training can be readily applied during the hectic workload. Moreover, the lack of a standardized protocol and appropriate storage locations for specimens collected for forensic purposes is a critical issue in an ED.

In addition, there is always the suspicion that violence in general is not yet fully perceived by professionals as a real clinical problem comparable to other diseases. This may result in less attention being paid to patient assessment. Moreover, interpersonal violence includes many different forms of violence that are interpreted by the general population as more or less severe. This may, for example, lead physicians to give different attention to a case of sexual abuse or intimate partner violence than to a street brawl. Finally, although some flow charts have recently been presented in the literature on the forensic measures to be followed when assessing victims of violence in the emergency department [7], and numerous guidelines exist, particularly by the European Council of Legal Medicine (ECLM) for specific types of violence (e.g. sexual violence [15] and elder abuse [16]), there are no internationally accepted protocols yet. Consequently, some questions may be forgotten during history taking and some details may be overlooked during lesion assessment. However, it should be borne in mind that the development of internationally recognized guidelines can be difficult due to the different legal and healthcare systems in individual countries. It should therefore be the task of national forensic departments to implement optimal guidelines for their country, focusing specifically on the clinical forensic medicine procedures, which are still too overlooked, at least in Italy. Therefore, it is important to remember that, particularly in the context of physical assault and violence, a series of clinical-legal medical examinations should be conducted to gather all relevant information about the aggression (i.e., timing and dynamics), describe the injuries, take photographs and specimens, prescribe imaging, and report to the judicial authority as appropriate [17]. A good description and photographs can “freeze the time” and show what happened, even after the injury has healed. A clear and unambiguous description should include several characteristics: type of injury (contusions, lacerations, abrasions, burns, puncture wounds, etc.), color [18], size and shape, location (including position and orientation in relation to body parts and anatomic body axes), number of lesions and aging (recent or old) [19–21]. Finally, timely examination may prove critical in the differential diagnosis between an accidental and nonaccidental injury. By performing all these task correctly, the patient receives the best clinical treatment and at the same time all the elements are provided that form the core of forensic evidence for possible later court proceedings [17]. These are considerations that may seem trivial, but data from the literature seem to indicate that these seemingly familiar concepts are not successfully applied in daily practice in the clinical setting [5]. Indeed, recent literature indicates that emergency physicians lack competence and practice in documenting, collecting, and preserving forensic evidence in many cases worldwide [3–5, 7]. Furthermore, because each type of violence has its own causes, risk factors, and health consequences including lethal ones (as in the case of child abuse [22, 23] or femicide [24, 25], it should begin to be considered at the same level as any other disease.

Therefore, there is an urgent need to train ED physicians on how to conduct a clinical forensic evaluation and appropriate legal procedures for victims of interpersonal violence to promote social justice, and assist communities in sentencing offenders [26]. A review conducted in 2021 comparing the effectiveness of education courses to improve the recognition and management of violence against women in couple relationships (Intimate Partner Violence, IPV) concluded that training can, to some extent, improve awareness and knowledge of IPV and increase the readiness of health workers to respond appropriately to these cases [27]. Nevertheless, there is not yet an appropriate training model that has been shown to be fully effective, as there are still significant gaps in development [28]. This applies all the more to physical violence in general, for which the literature on the training of ED physician is still very sparse.

There would be many ways to bring medico-legal expertise into emergency department setting and to convey the idea that violence should not only be treated clinically, but also prosecuted legally and prevented socially.

First, the study of clinical forensic medicine should also be introduced and taught more intensively at undergraduate level in order to start a satisfactory education at an early stage. A study by the European Council of Legal Medicine (ECLM) examined the level of training in forensic medicine in ECLM member countries and found that it is often very poor. Therefore, a standardized and appropriate undergraduate curriculum in forensic medicine adapted to local requirements was proposed [29].

Another possibility would be to implement an educational program that integrates awareness of violence and how to properly deal with it into emergency medicine residency training. In the Department of Emergency Medicine at the College of Louisville School of Medicine, Kentucky, USA, such a strategy has greatly improved the ability of emergency medicine residents to recognize, document, and address the forensic needs of their patients who are victims of violent and nonfatal trauma [30]. A study by the Australasian College for Emergency Medicine (ACEM) comes to a similar conclusion [31].

It would also be necessary to offer regular training and continuing education courses to the clinicians in ED to keep them up to date, including e-learning and digital training other than in-person lessons given the time constraints they have.

Otherwise, we argue that the introduction of forensic professionals in the emergency department would certainly be a better way to address this problem [32, 33]. In recent years, the role of the forensic nurse has become increasingly important as it is their task to take care of victims of violence by conducting a forensic external examination and collecting evidence, while showing sensitivity towards the victims [34, 35]. Therefore the forensic nurse could significantly improve the treatment of victims of violence by providing an important support for forensic medicine specialists who can be directly integrated into the emergency department or called in as consultants (e.g. as is the case with cardiologists or neurologists) when injured patients are admitted to the ED. Given the high caseloads in emergency departments and the fact that emergency physicians are busy with non-forensic clinical tasks (and rightly so), it should be ensured that there are dedicated forensic clinical staff [33, 36]. This solution has already been introduced through reforms and investments in some countries such as France, where this clinical service is already present in all hospitals [37].

### Limitations and future prospective

This was a monocentric study in which the analyses were performed in a single emergency department of a single hospital. Therefore, the results may not be generalizable to other populations or settings, as the population may not be representative, or the study design may not be appropriate for other settings. However, they may be useful for generating hypotheses and exploring new areas of research. In addition, some patients victims of violence may have not been recognized as such by their treating physicians. However, this should be considered an overriding limitation of the current organization rather than a limitation of the study. Indeed, the focus was on evaluating the performance of emergency physicians in medicolegal interventions in dealing with claimed cases of violence. Finally, with the exception of three cases in which photographs of the lesions were available, it was not possible in the other cases to assess retrospectively whether the description of the lesion was actually formally correct. This may seem like a limitation, but it should be considered that a doctor's description of an injury is equivalent to a medical certification and is reliable evidence in court even without a photograph.

This pilot study opens many possibilities for future in-depth studies, including the evaluation of physicians' performance broken down by the type of violence to highlight any shortcomings and standardize procedures for all cases of violence. In addition, it would be interesting to conduct multi-centre studies to compare different experiences and implement different training strategies to investigate which might prove most effective over time.

## Conclusions

A six-hours course has proven to increase the performance and the medico-legal skills of ED physicians in assessing and managing cases of interpersonal violence. A training course for emergency doctors on clinical forensic medicine may prove to be a good starting point and a good step forward, but as a stand-alone measure, it most likely will improve physician behavior only partially. To date, for emergency departments that do not have forensic personnel, regular training courses for ED physicians remain one of the few viable solutions.

## Data Availability

All the data have been reported in the manuscript.
